# Scalp ripples as prognostic biomarkers of epileptogenicity in pediatric surgery

**DOI:** 10.1002/acn3.50994

**Published:** 2020-02-25

**Authors:** Eleonora Tamilia, Matilde Dirodi, Michel Alhilani, P. Ellen Grant, Joseph R. Madsen, Steven M. Stufflebeam, Phillip L. Pearl, Christos Papadelis

**Affiliations:** ^1^ Laboratory of Children’s Brain Dynamics Division of Newborn Medicine Department of Medicine Boston Children's Hospital Harvard Medical School Boston Massachusetts; ^2^ Fetal‐Neonatal Neuroimaging and Developmental Science Center Division of Newborn Medicine Department of Medicine Boston Children’s Hospital Harvard Medical School Boston Massachusetts; ^3^ G. Tec Medical Engineering GmbH Guger Technologies OG Graz Austria; ^4^ Division of Epilepsy Surgery Department of Neurosurgery Boston Children’s Hospital Harvard Medical School Boston Massachusetts; ^5^ Athinoula A. Martinos Center for Biomedical Imaging Massachusetts General Hospital Harvard Medical School Boston Massachusetts; ^6^ Division of Epilepsy and Clinical Neurophysiology Department of Neurology Boston Children’s Hospital Harvard Medical School Boston Massachusetts; ^7^ Jane and John Justin Neurosciences Center Cook Children's Health Care System Fort Worth Texas; ^8^ School of Medicine Texas Christian University and University of North Texas Health Science Center Fort Worth Texas; ^9^ Department of Bioengineering University of Texas at Arlington Arlington Texas

## Abstract

**Objective:**

To assess the ability of high‐density Electroencephalography (HD‐EEG) and magnetoencephalography (MEG) to localize interictal ripples, distinguish between ripples co‐occurring with spikes (ripples‐on‐spike) and independent from spikes (ripples‐alone), and evaluate their localizing value as biomarkers of epileptogenicity in children with medically refractory epilepsy.

**Methods:**

We retrospectively studied 20 children who underwent epilepsy surgery. We identified ripples on HD‐EEG and MEG data, localized their generators, and compared them with intracranial EEG (icEEG) ripples. When ripples and spikes co‐occurred, we performed source imaging distinctly on the data above 80 Hz (to localize ripples) and below 70 Hz (to localize spikes). We assessed whether missed resection of ripple sources predicted poor outcome, separately for ripples‐on‐spikes and ripples‐alone. Similarly, predictive value of spikes was calculated.

**Results:**

We observed scalp ripples in 16 patients (10 good outcome). Ripple sources were highly concordant to the icEEG ripples (HD‐EEG concordance: 79%; MEG: 83%). When ripples and spikes co‐occurred, their sources were spatially distinct in 83‐84% of the cases. Removing the sources of ripples‐on‐spikes predicted good outcome with 90% accuracy for HD‐EEG (*P* = 0.008) and 86% for MEG (*P* = 0.044). Conversely, removing ripples‐alone did not predict outcome. Resection of spike sources (generated at the same time as ripples) predicted good outcome for HD‐EEG (*P* = 0.036; accuracy = 87%), while did not reach significance for MEG (*P* = 0.1; accuracy = 80%).

**Interpretation:**

HD‐EEG and MEG localize interictal ripples with high precision in children with refractory epilepsy. Scalp ripples‐on‐spikes are prognostic, noninvasive biomarkers of epileptogenicity, since removing their cortical generators predicts good outcome. Conversely, scalp ripples‐alone are most likely generated by non‐epileptogenic areas.

## Introduction

For patients with medically refractory epilepsy, resective surgery is the best treatment to gain seizure freedom.^1^ To be successful, epilepsy surgery requires the presurgical delineation of the epileptogenic zone (EZ), that is, the brain area indispensable for generating seizures.^2^ High‐frequency oscillations (HFOs; ripples: >80 Hz; fast‐ripples: >250 Hz) are promising interictal biomarkers of the EZ,^3^, ^4^, ^5^, ^6^ independently of conventional interictal spikes.^7^, ^8^, ^9^ Due to their low amplitude, HFOs are typically investigated using intracranial Electroencephalography (icEEG). Yet, icEEG recordings are not always performed prior to surgery since they present the risks of an invasive procedure and offer limited brain coverage. The possibility to record HFOs noninvasively through full‐head techniques would expand their use to an earlier stage in the presurgical epilepsy workup prior to icEEG.

Increasing evidence showed that HFOs below 250 Hz (ripples) can be seen on scalp EEG^10^, ^11^, ^12^, ^13^, ^14^, ^15^, ^16^, ^17^ and magnetoencephalography^18^, ^19^, ^20^, ^21^, ^22^, ^23^, ^24^, ^25^, ^26^, ^27^ (MEG) recordings, despite the small extent of their cortical generators.^11^, ^16^, ^28^ Yet, their translation to clinical practice is still limited. This may be attributed to the lack of studies that elucidate the spatial relationship between the gold standard, typically defined by icEEG ripples, and the generators of scalp‐recorded ripples, which can be localized noninvasively through electric or magnetic source imaging (ESI/MSI). Furthermore, very few studies investigated the localizing value of scalp‐recorded ripples as epilepsy biomarkers for surgery. This may have significant clinical impact since several icEEG studies showed that ripples are not always generated by epileptogenic areas, but can also represent physiological events generated by non‐epileptogenic tissues.^3^, ^29^, ^30^, ^31^

Previous studies showed that noninvasively localized ripple generators correlate with regions generating interictal spikes^22^, ^27^ or with different approximations of the EZ defined by clinical information.^14^, ^19^, ^22^, ^23^, ^25^, ^26^, ^27^ However, most of these studies included patients who did not necessarily undergo surgery, with the exception of two MEG studies on patients with focal MRI lesions^25^ or insular epilepsy.^26^ Given this paucity of noninvasive studies on surgical cohorts, the prognostic value of scalp‐recorded ripples for epilepsy surgery, as yet, remains largely unknown, particularly in children. An additional aspect that remains unclear is whether the clinical relevance of scalp‐recorded ripples is weakened by physiological oscillations. Although it is known that ripples co‐occurring with spikes represent the most pathological oscillations on icEEG (when compared to ripples independent from spikes),^32^ it is rather unknown whether this is also valid for scalp‐recorded ripples. Noninvasive studies showed that ripples are often seen at the same time as spikes,^23^, ^24^, ^33^ but it is uncertain whether such temporal concurrence reflects a common cortical source underlying the two biomarkers, or two separate generators, which are spatially distinct albeit active simultaneously.

This study aims to: (1) assess the ability of high‐density EEG (HD‐EEG) and MEG to localize ripples with respect to the intracranial gold standard (i.e., icEEG ripples), (2) distinguish between scalp ripples temporally co‐occurring with spikes (ripples‐on‐spike) and independent from spikes (ripples‐alone), and (3) evaluate their localizing value as biomarkers of the EZ in children with refractory epilepsy. We hypothesized that noninvasively localized ripples are concordant with icEEG ripples and ripples‐on‐spikes are better biomarkers of the EZ compared to ripples‐alone. To test our hypotheses, we localized interictal ripples using simultaneously recorded HD‐EEG and MEG data from children with refractory epilepsy, and compared them with icEEG ripples (from separate long‐term monitoring), spike sources, and resection.

## Methods

### Patients

We retrospectively reviewed patients with refractory epilepsy, who underwent epilepsy surgery at Boston Children’s Hospital between June 2011 and July 2016. We included patients who had: (1) preoperative HD‐EEG and MEG recordings; (2) long‐term icEEG monitoring with subdural electrodes and sampling frequency ≥ 600 Hz; (3) follow‐up after ≥ 1 year; and (iv) postimplantation computerized tomography (CT), preoperative, and postoperative MRI. Patients were excluded if <5 min of good‐quality data in ripple frequencies were available for HD‐EEG or MEG. Study protocol received approval by the Institutional Review Board of Boston Children’s Hospital (IRB‐P00022114; PI: C. Papadelis), which waived the need for written informed consent due to retrospective nature.

### Simultaneous HD‐EEG/MEG recordings

HD‐EEG/MEG recordings were conducted at the MEG Core Laboratory of Athinoula Martinos Center for Biomedical Imaging (Charlestown, MA), in a three‐layer magnetically shielded room (Imedco, Hägendorf, Switzerland) with a whole‐head 306‐channel MEG system (VectorView, Elekta Neuromag, Finland), consisting of 102 sensor units (two planar gradiometers and one magnetometer each). HD‐EEG was recorded using a nonmagnetic 70‐channel electrode cap (EASYCAP Brain Products, Herrsching, Germany) and two temporal electrodes (T1/T2). Online low‐pass infinite impulse response (IIR) filter of sixth order at 400 Hz was used at the time of the acquisition. To determine the head location with respect to the MEG sensors, four head position indicator coils were placed. A 3D digitizer determined the locations of the head position indicator coils and EEG electrodes with respect to anatomical landmarks on the head. Additional electrodes were placed to measure electrocardiography, electrooculography, and electromyography. Patients were instructed to rest or sleep during the recording. Spontaneous HD‐EEG/MEG data were recorded for 10‐12 sessions (4‐minute per session; sampling rate ≥ 600 Hz) as described elsewhere.^27^, ^34^ We analyzed data from three sessions that had been regarded as containing considerable interictal activity by the attending epileptologist, independently from this study, regardless of the patient’s vigilance state.^19^, ^23^, ^25^, ^26^ Intervals with artifacts or technical disruptions were excluded.

### Ripple Detection on HD‐EEG and MEG

Ripple detection was performed, separately on HD‐EEG and MEG, via preliminary automated detection followed by human visual review for rejection of false positives and artifacts. HD‐EEG was analyzed on average montage.^24^, ^27^

#### Automated detection

**Figure 1:**
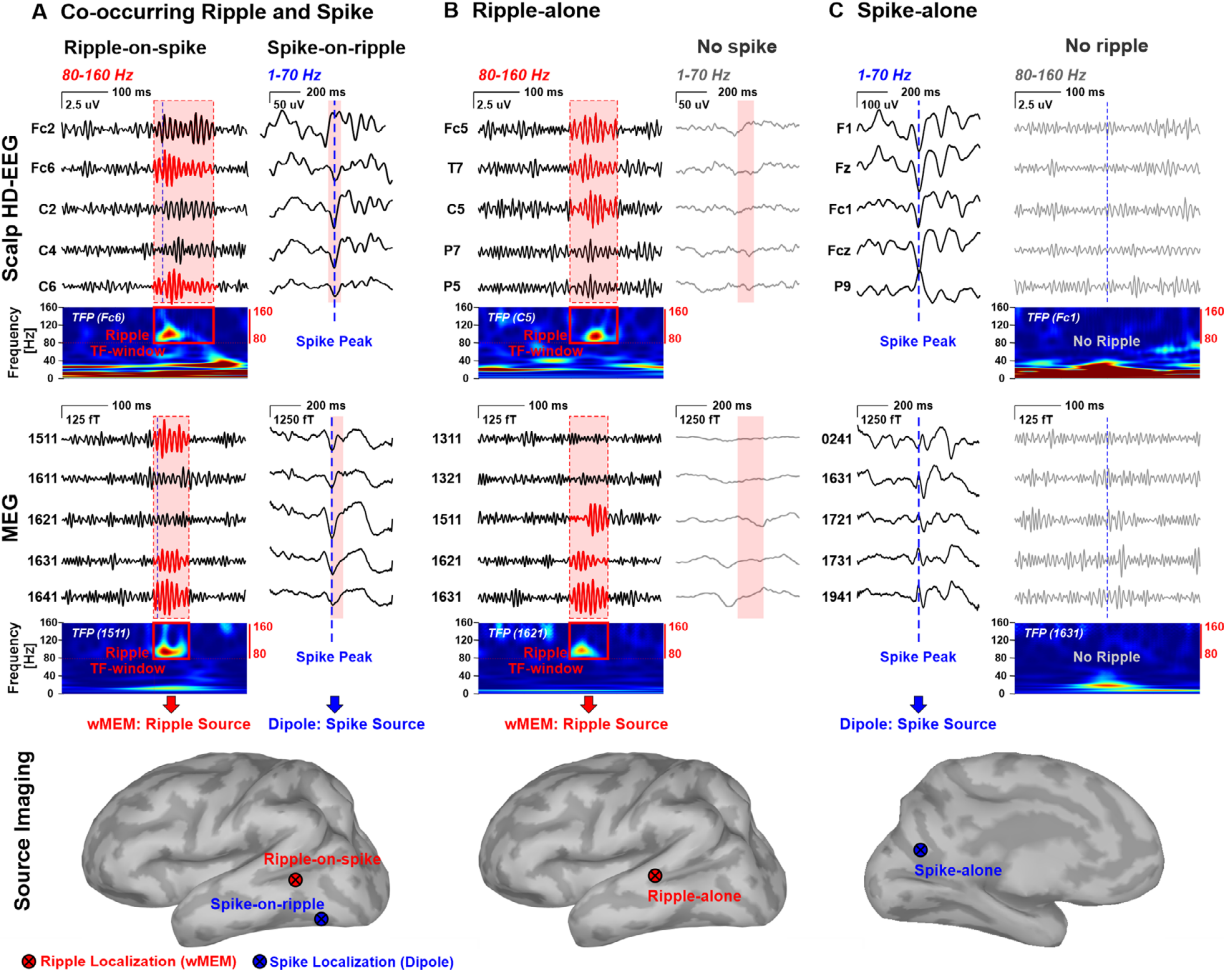
Types of Ripples and Spikes on HD‐EEG and MEG data and their Source Localization. Examples of interictal ripples and spikes on HD‐EEG or MEG data (from patient #9 and #11, respectively) with distinction between ripples and spikes that co‐occur in time (A), ripples‐alone (B), and spikes‐alone (C). Each scenario shows: (i) signals filtered between 80 and 160 Hz (for ripple visualization) on an expanded timescale, where ripple time windows are marked in red (when present, i.e., A and B); (ii) time‐frequency plane (TFP) of one of the channels; the red box (when present, i.e., A and B) marks the ripple time‐frequency window with isolated spectral peak; (iii) signals filtered between 1 and 70 Hz (for spike visualization), where dashed blue line (when present, i.e., A and C) marks the spike peak; and (iv) localization, on the patient’s 3D cortical surface, of the sources of each ripple (red dot) or spike (blue dot) seen on the scalp. For spikes and ripples that co‐occurred, localization was performed separately for spikes (dipole on 1–70 Hz data) and ripples (wMEM on 80‐160 Hz data), so that two independent generators (ripple‐on‐spike and spike‐on‐ripple) were localized. Cortical surface is displayed as inflated to show the cortical surface within the sulci. Electroencephalography (EEG) data are from a 9‐year‐old girl with right frontal Focal Cortical Dysplasia (FCD) (patient #9). MEG data are from a 10‐year‐old girl with left parietal FCD (patient #11).

We used a validated HFO detector,^3^ after parameters were adapted to ensure high sensitivity on HD‐EEG/MEG. The adapted algorithm detected individual ripples on 80–160 Hz band‐pass filtered (Finite Impulse Response) signals when: (1) the envelope’s z‐score was> 3 and < 12 (to exclude high‐amplitude noise or artifacts);^27^ (2) there were at least four oscillations lasting> 25 ms in total (i.e., 4∙1/160 Hz = 25 ms); and (3) time‐frequency plane (TFP) showed spectrally isolated ripple frequency components (to reject filtering effects)^3^, ^27^, ^35^. Frequencies above 160 Hz were excluded to ensure the highest signal‐to‐noise ratio, based on preliminary review of HD‐EEG/MEG data, and to set a cutoff frequency more than three times below our minimum sampling rate.^36^ A ripple time window was identified when one or more individual ripples were detected with overlapping duration (Fig. 1A–B). We will refer to ripple time windows as “ripples.” Ripples in> 75% of channels were rejected, since resembling muscle and movement artifacts.^37^

#### Visual review and artifact rejection

False positive detections were reduced through visual review of each ripple by two independent reviewers (E.T. and M.D.), experienced in HFO analysis and artifact recognition, blinded to patient’s outcome and resection. In case of disagreement, decision was taken after discussion. Each ripple was reviewed on 80–160 Hz filtered (3‐sec/page) and unfiltered data (10‐sec/page). We excluded artifacts following previous guidelines.^10^, ^11^, ^12^, ^27^ Events were discarded if they: (1) overlapped with cardiac beats on electrocardiography; (2) did not clearly stand out from surrounding background; or (3) showed very irregular morphology, high amplitude compared to background, or high amplitude/frequency variability.^10^ Electrooculography and electromyography data were reviewed, when available, to exclude artifacts.

### Categorization of Ripples and Spikes

Visual marking of interictal spikes (or sharp waves) was performed on 1–70 Hz data^34^ by C.P. and M.A blind to ripple detection and to patient’s outcome and resection. To reduce the possibility of missing spikes, additional marking was performed by reviewing each ripple time window. Co‐occurrence between ripples and spikes was defined when a spike peak fell within a ripple time window (with 20‐ms tolerance) even if in a different channel. Hence, each ripple was categorized as ripple‐on‐spike (Fig. 1A) or ripple‐alone (Fig. 1B). Similarly, spikes were distinguished in spikes‐on‐ripple (Fig. 1A) or spikes‐alone (Fig. 1C).

### Source imaging

ESI and MSI were performed independently for HD‐EEG and MEG. Cortical generators of ripples and spikes were localized separately, even when co‐occurring in time: ESI/MSI was performed distinctly on the 80–160 Hz filtered data (to localize the ripple) and 1–70 Hz filtered data (to localize the spike), as shown in Figure 1A.

#### Head model

We extracted individual cortical surfaces from the preoperative MRIs via *Freesurfer* 38 and constructed realistic head models using *OpenMEEG*.^39^ We used a three‐layer boundary elementary model for both HD‐EEG and MEG.^34^ Source space was constrained to the cortex.

#### Ripple sources

**Figure 2:**
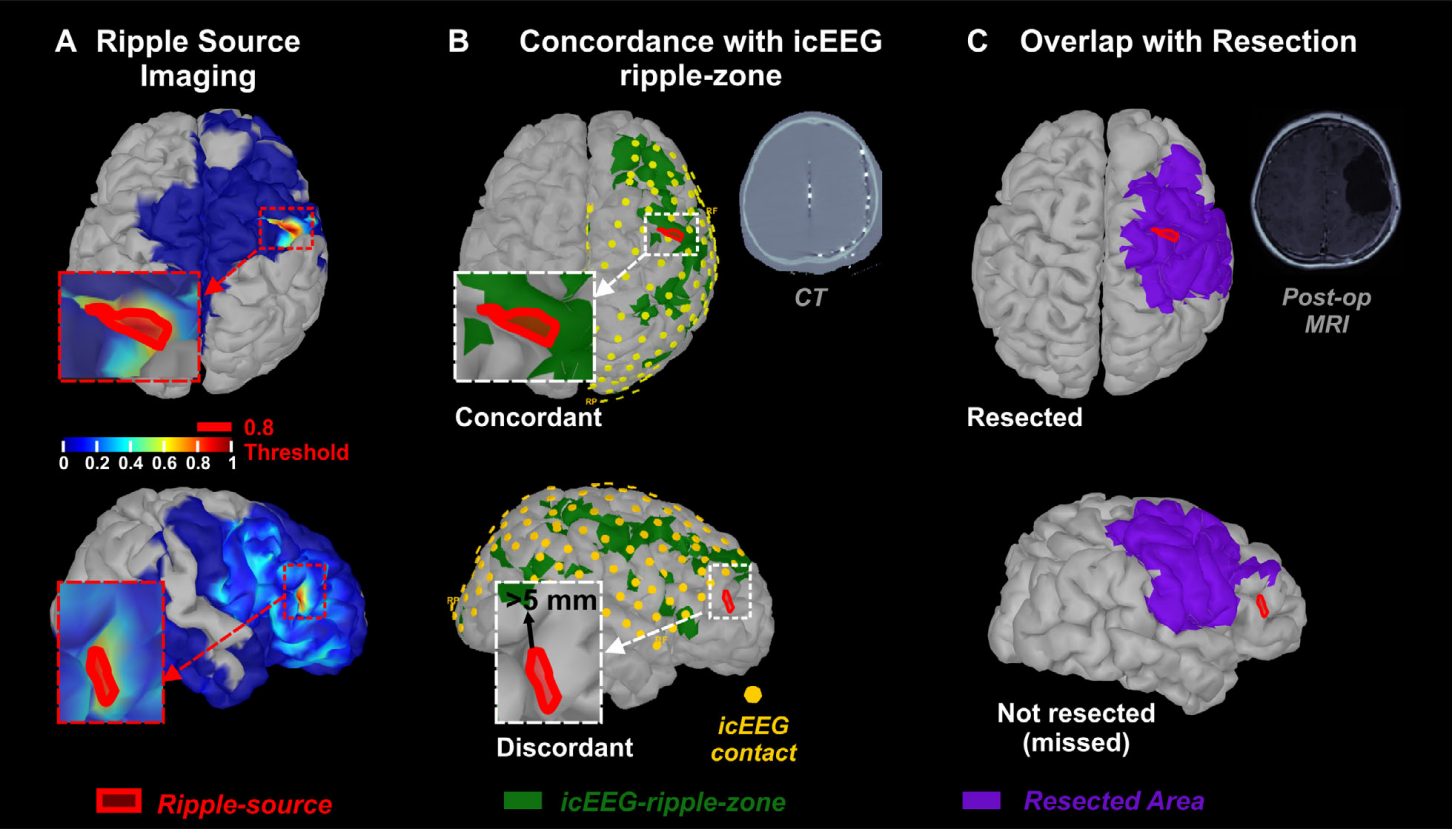
Comparison of Ripple Source Imaging with icEEG‐defined ripple‐zone and Surgical Resection. (A) Source imaging (wMEM) on two ripples noninvasively recorded via MEG from a 2‐year‐old boy with tuberous sclerosis complex (patient #5). Location of the ripple source (red area) was defined by the vertices with activation amplitude above 80% of the maximum (i.e., normalized value > 0.8). 3D cortical surface was extracted from patient’s preoperative MRI. (B) Comparison of ripple source (red area) with the icEEG‐defined ripple‐zone (green area). Contacts with normalized ripple rates below 0.2 were not included in icEEG‐ripple‐zone. Location of the icEEG contacts (yellow dots) on the cortical surface was defined by co‐registering preoperative MRI with postimplantation CT scan. Only ripple sources localized within the brain area covered by icEEG contacts were compared with the icEEG‐ripple‐zone. A Source within 5 mm from the icEEG‐ripple‐zone was regarded *concordant*, or *discordant* otherwise. (C) Comparison of ripple source (red area) with the surgically resected area (purple area) defined by co‐registering preoperative and postoperative MRI. Ripple sources within 5 mm from resection were regarded resected or not‐resected (or missed) otherwise. Classification of a ripple Source as *concordant/discordant* or *resected/missed* was based on the distance of the most active vertex or the majority (>50%) of its vertices.

ESI and MSI of ripples were performed on band‐pass filtered data (80‐160 HZ) using the wavelet Maximum Entropy on the Mean (wMEM)^40^ in *Brainstorm*,^41^ which allows localizing specific scales of interest. Data were down‐sampled to 640 Hz to ensure that the second scale corresponded to ripples (80–160 Hz).^19^, ^27^ Source localization was performed across each ripple time window. Diagonal noise covariance matrix was estimated from a 0.25–0.5 s window before or after the ripple. Given the scarcity of ripples on gradiometers, wMEM was performed only on magnetometers where most of the ripples were seen. Such difference may be likely due to the magnetometers’ higher sensitivity to fields originating within a wide distance,^42^, ^43^ which is particularly relevant in children, as their head is more likely to be distant from the MEG helmet than in adults. We obtained a wMEM map for each ripple, where activation values were associated to each cortical vertex and time point. Each wMEM map was averaged across time and normalized in amplitude (the most active vertex had a value equal to one).^19^, ^27^ The highest amplitude vertices (i.e., normalized amplitude > 0.8) delineated each ripple source (Fig. 2A).

#### Spike sources

We localized the source of each spike (spike source) with an equivalent current dipole, the only conventional method approved for clinical practice.^44^ Source analysis was performed on 1–70 Hz filtered data at the spike peak using dipole scanning.^34^ For spikes and ripples that co‐occurred (Fig. 1A), localization was performed separately for spikes (dipole on 1–70 Hz data) and ripples (wMEM on 80–160 Hz data), so that two independent generators (ripple‐on‐spike and spike‐on‐ripple) were localized. Then, we assessed whether the two co‐occurring biomarkers had *distinct* or *common* generators: we regarded as spatially *distinct* any source of ripples‐on‐spikes that was> 15‐mm apart from any source of spikes‐on‐ripple, or *common* otherwise.

### Concordance with icEEG‐ripple‐zone

The location of each icEEG contact was drawn on the presurgical MRI co‐registered with postimplantation CT, and mapped on the 3D cortical surface (Fig. 2B). The area covered by icEEG (icEEG coverage) was defined by the cortical vertices within 10 mm from any icEEG contact. Ripple sources were classified as *covered*, when their minimum distance (Euclidean) from the icEEG coverage was <5 mm (for the most active or the majority of its vertices), or *uncovered* otherwise. *Uncovered* sources were excluded from validation against icEEG‐ripple‐zone.

To define the icEEG‐ripple‐zone, we analyzed 5–10 min of interictal icEEG data, recorded using XLTEK NeuroWorks (Natus Medical Inc.), as previously described.^3^ We detected ripples using a validated automated detector.^3^ The icEEG‐ripple‐zone was defined by assigning to each vertex within icEEG coverage the value of the closest icEEG contact ripple rate (normalized between 0 and 1). Then, ripple sources were classified as *concordant,* if their distance from the icEEG‐ripple‐zone was <5 mm, or *discordant* otherwise (Fig. 2B). Finally, we calculated the percentage of icEEG ripples recorded from areas where ripple sources were localized to estimate the sensitivity of ripple source imaging to icEEG ripples.

### Resection and Outcome

The resection was prospectively tailored for each patient, independently from this study. Resection margins were delineated on the presurgical MRI after co‐registration with postsurgical MRI^34^ and then projected to the 3D cortical surface (Fig. 2C), that is, to the closest vertices within 10 mm. Each ripple source was classified as *resected,* if its minimum distance from the resected area (D_RES_) was <5 mm or *missed* otherwise (Fig. 2C). Similarly, D_RES_ was calculated for spike sources, which were classified as *resected* or *missed*.

Postsurgical outcome was determined based on the latest follow‐up visit using Engel classification^45^ and dichotomized into good outcome, that is, Engel 1, and poor outcome, Engel ≥ 2.

### Outcome prediction

To assess the clinical utility of the two types of ripples (i.e., ripples‐on‐spike or ripples‐alone) in terms of individualized patient care for guiding surgery, we evaluated whether removing their sources predicted postsurgical outcome. Receiver operating characteristic (ROC) curves were built, for ESI and MSI separately, to test the ability of ripples to predict outcome based on the number of missed sources during resection. We considered good outcome following resection to be the ground truth, that is, unambiguous proof of EZ resection. We regarded as: (1) true positives (TP), good outcome patients with low number of missed sources (i.e., complete resection); (2) true negatives (TN), poor outcome patients with high number of missed sources (i.e., incomplete resection); (3) false positives (FP), poor outcome patients with complete resection; and (4) false negatives (FN), good outcome patients with incomplete resection. We estimated positive predictive value [PPV = TP/(TP + FP)], negative predictive value [NPV = TN/(TN + FN)], and F‐measure (or accuracy) [2TP/(2TP + FP+FN)] to assess prediction performance. The value that provided the highest F‐measure determined the optimal threshold to define low or high number of missed sources (i.e., complete or incomplete resection). The same analysis was performed to evaluate whether resection of spike sources (spikes‐on‐ripples or spikes‐alone) predicted outcome.

### Statistical analysis

Wilcoxon rank‐sum test was used to compare continuous variables between good‐ and poor outcome groups. Rates of ripples and spikes were compared using a z‐test of the Poisson event‐rate difference.^46^ Kruskal‐Wallis analysis of variance was used to compare resection distance between interictal events and modality (ESI vs. MSI), with Bonferroni correction for post hoc tests. Nonparametric statistics were used since variables were not normally distributed based on Kolmogorov‐Smirnov test. Proportions were compared using χ^2^ test. Fisher’s exact test determined whether the presence of missed sources (above threshold) was associated with outcome. *P* **‐**values < 0.05 were considered significant. Results are expressed as median (inter‐quartile range). MATLAB 2018a (The MathWorks, Inc) was used for statistical analysis.

## Results

### Patients

Twenty patients met the inclusion criteria and were initially included. Three of them were then excluded due to continuous artifacts and/or low signal‐to‐noise ratio in ripple frequencies (>80 Hz) on HD‐EEG or MEG data. Table 1 reports patients’ clinical findings and demographics. Eleven patients were Engel 1 after surgery (median follow‐up: 29 months), while the remaining were Engel 2 or 3 (median follow‐up: 28 months). Median age was 12.3 (8.9–14.7) years, without difference between good‐ and poor outcomes (*P* = 0.9). Five patients were MRI‐negative (nonlesional) (see Table 1). Resected area was 131 (121–169) cm^2^ and 77 (49–251) cm^2^ in good‐ and poor outcome patients (*P* = 0.3).

**Table 1:** Demographics, clinical characteristics, and ripple detections of all patients.

Pt/ Sex	Age [yrs]	Age at Epilepsy Onset [yrs]	MRI	icEEG contacts [#] (Lobes)	Resection Lobe	Pathology	Engel (f/u mo.)	SamF [Hz]	Scalp‐Recorded Ripples					
									Detected [#]				Distinct Ripple source^a^ [%]	
									HD‐EEG		MEG		*ESI*	*MSI*
									*on‐SP*	*alone*	*on‐SP*	*alone*		
1/ M	8.7	4	NEG	80 (F,T)	F,P	NS	1 (44)	600	18	3	12	0	22	100
2/ Fe	10.3	9	DNET	144 (F,P,IH)	F,P	DNET	1 (31)	600	4	1	0	1	100	n/a
3/ Fe	13.5	10	NEG	72 (FT)	T	FCD	1 (48)	1000	4	5	2	0	75	100
4/ M	13.0	9	Neoplasm	72 (FT)	T	GAN	1 (40)	600	5	0	1	0	100	100
5/ M	1.8	0	TSC	112 (F,P,IH)	F	Tuber	1 (41)	1000	14	3	12	4	50	42
6/ Fe	17.8	15	NEG	88 (T,sub‐F)	T	NS	1 (29)	600	7	7	2	1	86	50
7/ M	15.4	4	NEG	88 (F,T,O)	T	FCD	2 (25)	600	11	2	1	1	100	100
8/ M	14.8	4	NEG	88 (T,P, post‐F)	F	NS	3 (36)	600	8	5	2	1	75	100
9/ Fe	8.4	1	FCD	112 (F,P,IH)	F	FCD	1 (42)	600	21	44	2	6	90	100
10/ Fe	14.7	6	FCD	72 (F,P,T)	P	FCD	1 (26)	2035	12	7	4	2	100	50
11/M	11.8	8	Encephalomalacia	72 (F,P,T)	T,P	NS	1 (12)	1000	4	3	6	0	75	83
12/M	12.3	7	FCD	112 (F,T)	F,T	FCD	3 (39)	1000	20	0	1	0	45	0
13/M	12.3	0	L MCA infarct	136 (F,T,P,O)	F,T	FCD	2 (37)	1000	16	10	20	3	88	75
14/M	9.9	7	PMG	64 (F,T,ant‐P)	P,O	FCD	1 (12)	1000	22	4	5	0	82	40
15/ Fe	8.9	9	FCD/Encephalitis	86 (F,P,T)	F,P	Encephalitis	3 (25)	600	109	38	85	8	9	15
16/ Fe	6.4	4	FCD	72 (F,T)	F Op	FCD	2 (31)	1000	28	14	7	3	93	100
17/M	17.5	5	FCD	64 (F)	F	FCD	1 (28)	1000	0	0	0	0	n/a	n/a

*Pt, *Patient; *yrs*, years; *on‐SP, *ripples that co‐occurred in with a spike; *Fe*, Female; *M*, Male; *NEG*, Negative; DNET, Dysembryoplastic Neuroepithelial Tumor; *TSC*, Tuberous Sclerosis Complex; *FCD*, Focal Cortical Dysplasia; *MCA*, middle cerebral artery, *PMG*: Polymicrogyria; *L*, Left; *R*, Right; *F*, Frontal; *T*, Temporal; *P*, Parietal; *O*, Occipital; *IH, *Inter‐Hemisphere; *NS*, Nonspecific; *GAN*, Ganglioma; *f/u*, follow‐up; *Op*, operculum; *samF*, sampling frequency.

a Percentage of ripples on spikes whose generators (wMEM localization) are spatially distinct from concurrent spike generators (dipoles).

### Ripples on HD‐EEG and MEG

Sixteen patients showed ripples on HD‐EEG and MEG. Patient #17 did not show scalp ripples and was excluded from further analysis. We found 449 ripples on HD‐EEG and 192 on MEG (where “ripples” refer to ripple time windows) with higher rates on HD‐EEG than MEG (1.5 vs. 0.46 ripples/min; *P* = 0.005). Of all HD‐EEG ripples (*n* = 449), 19% (*n* = 86) were also seen on MEG, while 81% only on HD‐EEG. For MEG ripples (*n* = 192), 45% (*n* = 86) were also seen on HD‐EEG, while 55% only on MEG.

### Concurrence between Ripples and Spikes

**Figure 3:**
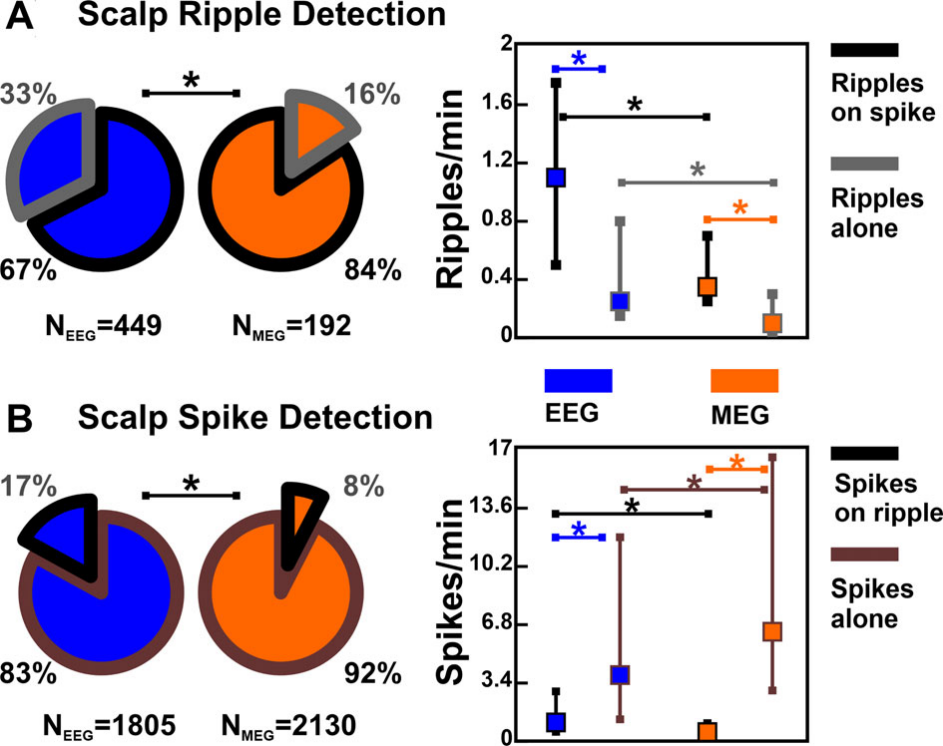
Detection of Ripples and Spikes on HD‐EEG and MEG. Significant differences (*P* < 0.05) are marked by an asterisk (*). (A) *Left*: Total number of ripples detected on MEG (orange) and HD‐EEG (blue) and percentage of ripples‐on‐spike (wedge with black border) versus ripples‐alone (grey border). *Right*: Medians (square) and inter‐quartile ranges (whiskers) of the different types of noninvasively detected ripples. (B) *Left*: Total number of spikes detected on MEG (orange) and HD‐EEG (blue) and percentage of spikes‐on‐ripple (wedge with black border) versus spikes‐alone (grey border). *Right*: Medians and inter‐quartile ranges of the different types of noninvasively detected spikes.

Most of the scalp ripples co‐occurred with spikes (Fig. 3A): 67% for HD‐EEG (*n* = 303) and 84% for MEG (*n* = 162). Table 1 reports the numbers of ripples per patient. Higher rates of ripples were found on HD‐EEG than MEG (Fig. 3A; ripples‐on‐spike: 1.08 vs. 0.25 ripples/min; *P* = 0.002; ripples‐alone: 0.38 vs. 0.08 ripples/min; *P* < 0.001).

Spikes were found in all patients with similar rates between HD‐EEG and MEG (4.8 and 6.7 spikes/min, *P* = 0.2, Fig. 3B), which were higher than ripple rates (*P* < 0.001). Rates of spikes‐alone were higher than spikes‐on‐ripple (HD‐EEG: 3.83 vs. 1.08 spikes/min, *P* < 0.001; MEG: 6.33 vs. 0.25 spikes/min, *P* < 0.001).

Examining ripples and spikes that co‐occurred (independently localized), we found that 84% (56‐98%) of the ripple sources for ESI and 83% (42–100%) for MSI were spatially distinct from spike sources (Table 1 reports percentages per patient), without differences between lesional and nonlesional cases (HD‐EEG: *P* = 0.6; MEG: *P* = 0.1).

### Concordance of Ripple Sources with icEEG

**Figure 4:**
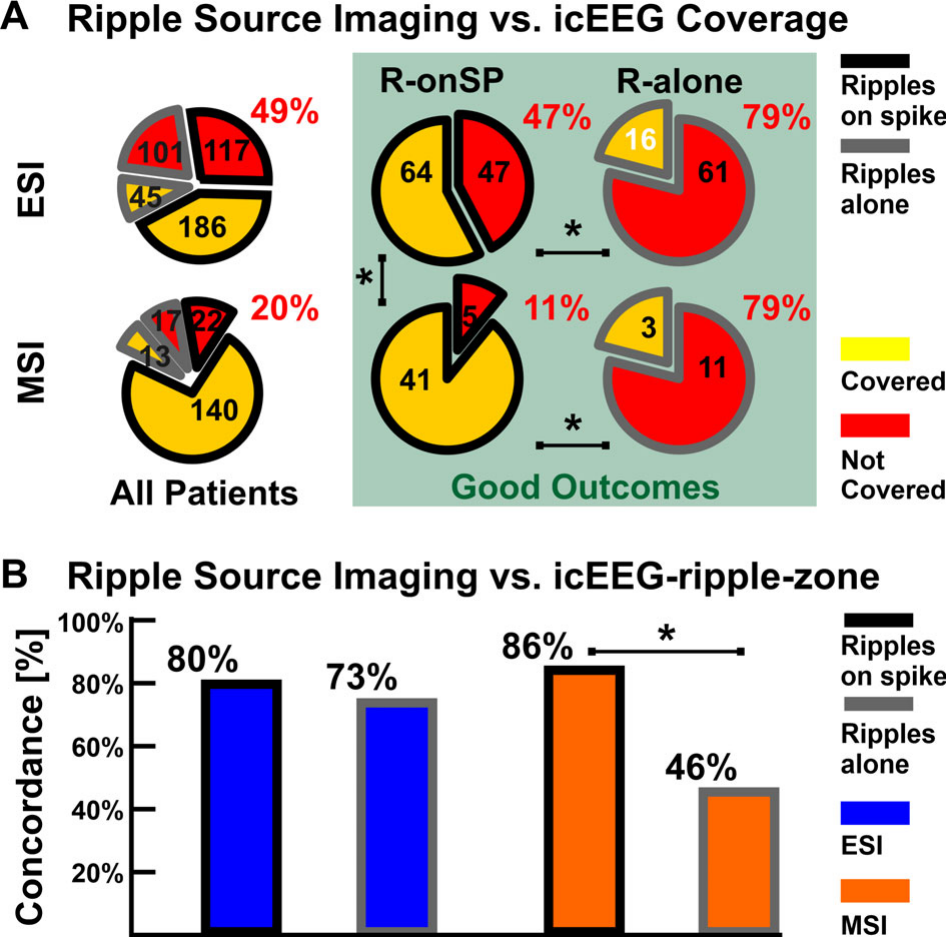
Validation of Ripple Source Imaging against icEEG. Significant differences (*P* < 0.05) are marked by an asterisk (*). (A) Proportion of ripple sources covered by icEEG contacts (yellow wedges) or not covered (red wedges) by ESI (top) and MSI (bottom), separated into ripples‐on‐spikes (black border) and ripples‐alone (grey border). Green frame reports the results restricted to good outcome patients. (B) Proportion of ripple sources concordant with icEEG‐ripple‐zone.

The icEEG coverage included an average of 87 (72‐112) subdural contacts per patient. The proportion of ripple sources localized within icEEG coverage was lower for ESI (51%; *n* = 231) than MSI (80%; *n* = 80; Fig. 4A). In good outcome patients, who are demonstrative of successful resection and optimal icEEG placement, 79% of ripples‐alone were *uncovered*, both for ESI and MSI (Fig. 4A). Conversely, for ripples‐on‐spikes, most of the ripple sources were covered by icEEG (ESI: 53%; MSI: 89%, *P* < 0.001).

All patients showed icEEG ripples. The icEEG‐ripple‐zone included 33 (22‐41) contacts with mean rate of 9.8 (6.3–14.3) ripples/min. Of all the ripple sources within icEEG coverage, 79% for ESI and 83% for MSI were concordant to the icEEG‐ripple‐zone, without difference between lesional and nonlesional patients (ESI: *P* = 0.8; MSI: *P* = 0.5). For MSI, concordance was higher for ripples‐on‐spikes than ripples‐alone (86% vs. 46%; *P* < 0.001), while ESI showed no difference (80% vs. 73%, Fig. 4B). No difference was seen between MSI and ESI for ripples‐on‐spikes (*P* = 0.1). Sensitivity of ESI and MSI to icEEG ripples was 32% and 23%, respectively.

### Overlap with resection and outcome

**Figure 5:**
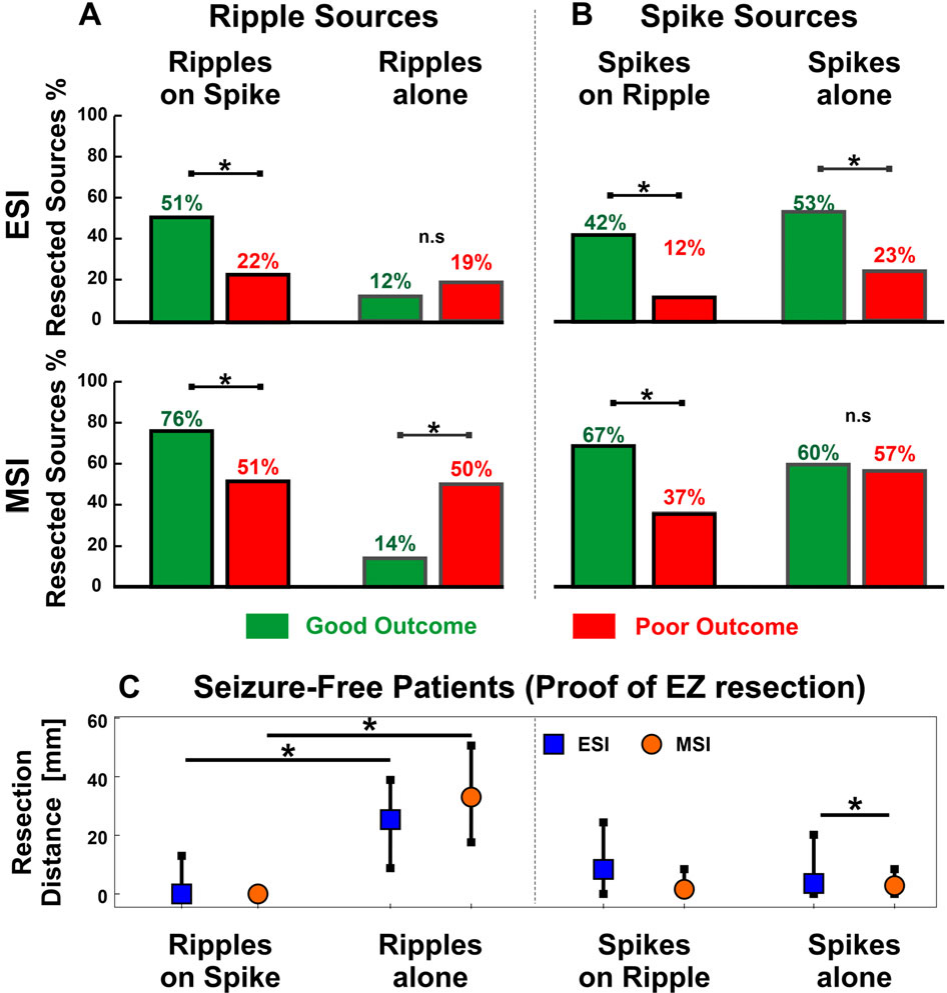
Overlap of Ripple and Spike Sources with Resection. (A–B) Proportions of ripple sources (A) and spike sources (B) localized inside the resected area in the good outcome (green bars) and poor outcome groups (red bars). (C) Distance of ripple sources (left) and spike sources (right) from the resected area in case of proof of EZ resection, that is, in good outcome patients (*n* = 10). Medians (square or dot) and IQRs (whiskers) are shown. Significant differences (*P* < 0.05) are marked by an asterisk (*).

For ripples‐on‐spikes, the proportion of resected ripple sources was higher in good‐ than in poor outcome patients (Fig. 5A; ESI: 51% vs. 22%, *P* < 0.001; MSI: 76% vs. 51%, *P* = 0.003), while this was not found for ripples‐alone (Fig. 5A).

For HD‐EEG spikes, a higher proportion was resected in good outcome compared to poor outcome patients (spikes‐on‐ripples: 42% vs. 12%, *P* < 0.001; spikes‐alone: 53% vs. 23%, *P* < 0.001; Fig. 5B). For MEG spikes, the proportion of resection was higher in good outcome than poor outcome patients for spikes‐on‐ripples (67% vs. 37%, *P* = 0.002, Fig. 5B), but not spikes‐alone (60% vs. 57%, *P* = 0.2).

In good outcome patients (Fig. 5C), sources of ripples‐alone were farther from resection than ripples‐on‐spikes (ESI: 25.4 mm vs. 0 mm, *P* < 0.001; MSI: 33 mm vs. 0 mm, *P* < 0.001). Conversely, no difference was found between spikes‐alone and spikes‐on‐ripples (ESI: 3.6 vs. 8.4 mm, *P* = 0.05; MSI: 2.8 vs. 2.1 mm, *P*≈1). Finally, the generators of ripples‐alone were farther from resection than spikes‐alone (ESI and MSI: *P* < 0.001), while when ripples and spikes co‐occurred, ripple sources were closer to resection than spike sources for ESI (*P* = 0.005), but not MSI (*P* = 0.4).

### Outcome prediction

Table 2 reports the ROC curve results. For ripples‐on‐spikes, the number of ripple sources that were missed during resection was predictive of outcome: incomplete resection of ripple sources predicted poor outcome using ESI (*P* = 0.008) and MSI (*P* = 0.044), with PPV of 90% and 75%, NPV of 83% and 100%, and accuracy of 90% and 86%, respectively. Conversely, incomplete resection of ripples‐alone did not predict outcome (ESI: *P* = 0.3; MSI; *P *≈ 1).

**Table 2:** Resection of ripple and spike sources and prognostic value for postoperative outcome.

Event	Event	Missed Sources [#] (median)		Missed Sources Thresh^a^	Resection	Patients [#]			PPV	NPV	F	MK^b^	Phi^c^	p^d^
		*Good*	*Poor*			*Tot*	*Good*	*Poor*						
EEG Ripples	*with spike*	54 (4.5)	159 (11)	9	*Incomplete*	6	1	5	90	83	90	73	0.73	0.008*
					*Complete*	10	9	1						
	*Alone*	68 (4)	56 (5)	12	*Incomplete*	8	4	4	83	50	67	33	−0.34	0.3
					*Complete*	6	1	5						
EEG Spikes	*with ripple*	64 (6)	162 (12.5)	16	*Incomplete*	3	0	3	77	100	87	77	0.62	0.036*
					*Complete*	13	10	3						
	*Alone*	396 (20.5)	463 (40)	32	*Incomplete*	9	4	5	86	56	71	42	0.42	0.2
					*Complete*	7	6	1						
MEG Ripples	*with spike*	11 (1)	57 (4)	7	*Incomplete*	3	0	3	75	100	86	75	0.61	0.044*
					*Complete*	12	9	3						
	*Alone*	12 (2)	8 (1)	2	*Incomplete*	5	3	2	40	40	40	−20	−0.2	1
					*Complete*	5	2	3						
MEG Spikes	*with ripple*	13 (1)	57 (2.5)	8	*Incomplete*	2	0	2	70	100	82	70	0.48	0.1
					*Complete*	13	9	4						
	*Alone*	384 (6.5)	286 (28.5)	7	*Incomplete*	11	5	6	100	55	67	55	0.52	0.09
					*Complete*	5	5	0						

a Number of missed sources that provided the best prediction performance (threshold used to define complete or incomplete resection).

b Markedness (MK) = PPV = NPV – 100 (measure of trustworthiness of positive and negative predictions).

c Phi Coefficient of Association.

d Fisher’s exact test.

* *P*‐value statistically significant (<0.05).

For HD‐EEG, incomplete resection of spikes‐on‐ripples predicted outcome with PPV of 77%, NPV of 100%, and accuracy of 87% (*P* = 0.036), while this was not found for spikes‐alone (*P* = 0.2; Table 2). Resection of any type of MEG spikes was not associated with outcome.

## Discussion

This study shows for the first time that noninvasive source imaging (via HD‐EEG or MEG) localizes ripples with high precision to the intracranial gold standard (icEEG ripples) in children with refractory epilepsy. Scalp‐recorded ripples that co‐occur with spikes (ripples‐on‐spike) are prognostic biomarkers of epileptogenicity, which provide nonredundant information about the EZ, contrary to scalp ripples‐alone, which more likely reflect physiological events. These interpretations are based on our main findings: (i) noninvasive ESI and MSI localize scalp ripples precisely compared to the conventional icEEG‐ripple‐zone; (ii) cortical generators of scalp‐recorded ripples and spikes that co‐occur in time are often spatially distinct; (iii) ripples‐alone on HD‐EEG or MEG are most likely localized outside epileptogenic areas compared to ripples‐on‐spikes; and (iv) missed resection of areas that generate scalp‐recorded ripples‐on‐spikes, but not ripples‐alone, predicts poor outcome.

### Scalp HD‐EEG and MEG Localize Precisely the Ripple Cortical Generators

We quantified the ESI/MSI ability to localize ripples with respect to the icEEG gold standard: HD‐EEG and MEG localized ripples with 79–83% precision when compared to the ripple‐zone delineated by the subdural contacts that recorded ripples. These data contribute to the ongoing debate on whether scalp‐recorded and icEEG ripples are expressions of the same underlying phenomenon by showing spatial consistency between the two. Such consistency indirectly suggests that HD‐EEG or MEG can record the same ripples that we typically record via icEEG. These results, taken together with previous findings,^11^ suggest that the notion that at least 4–10 cm^2^ of synchronously active cortex is necessary to observe epileptiform discharges on the scalp^47^, ^48^ does not hold for ripples.^28^ Moreover, icEEG studies showed that ripples are focal events that can appear in individual subdural contacts or be asynchronously present in different contacts.^3^, ^11^, ^49^ We thus may speculate that ripples on HD‐EEG or MEG reflect multiple focal generators asynchronously activated within a short latency, while focal ripples on individual icEEG contacts (which constitute the majority of icEEG ripples in children with refractory epilepsy)^3^ are most likely missed by HD‐EEG or MEG due to spatial undersampling.^11^ This may explain the low numbers of scalp‐recorded ripples and low sensitivity of noninvasive ripple source imaging to icEEG ripples (23–32%). Moreover, as in previous studies,^19^, ^23^ we recorded more EEG than MEG ripples. This may be attributed to the higher proximity of EEG sensors to the sources compared to MEG (especially in children when adult MEG systems are used), but also by the different sensitivity profiles of EEG and MEG:^50^, ^51^ MEG is blind to radial sources and less sensitive to deep sources, but more sensitive to environmental noise than HD‐EEG.

### Co‐occurring ripples and spikes on scalp reflect distinct underlying generators

A high proportion of ripples on HD‐EEG (67%) and MEG (84%) overlapped in time with spikes; although this high proportion may be biased by our selection of the most interictally active epochs, it is consistent with previous findings.^10^, ^23^ Since spike localization (via conventional dipoles) is well established in clinical practice compared to ripples, it is crucial to determine whether ripple hunting is worthwhile. Van Klink and colleagues^52^ showed that ripples could occur at the same time as a spike, but on a different channel, speculating on the presence of two different groups of cells being active to generate either ripples or spikes. Our data confirm their observation at the sensor level (see examples in Fig. 1A), but also reinforced it at the cortical level providing a novel piece of knowledge: ~80% of ripple cortical generators were spatially different (>15 mm away) from spike generators (conventional dipoles), although simultaneously active. This indicates that these two concurrent events often reflect distinct active sources. Thus, scalp ripples provide nonredundant localizing information on epileptogenicity, which adds to looking only at interictal spikes. Our data can be interpreted in light of previous icEEG findings, which showed that HFOs behave differently from spikes in response to antiepileptic drugs and after seizures,^53^ implying that they are generated by different pathophysiologic mechanisms even if co‐occurring in time. Although single neuron recordings (microwire electrodes) would be ideal to investigate underlying cellular mechanisms, our data provide some hints to this regard: scalp ripples and spikes possibly reflect distinct pathophysiological mechanisms generated by different epileptogenic networks (as demonstrated on icEEG),^3^, ^54^ whose activation is not independent to each other, but may be facilitated by a common brain state.^55^, ^56^ We also acknowledge that the use of different localization methods (dipoles vs. wMEM) may confound the localization comparison between spike and ripple generators; yet, we believe that their presence on different sensors strongly supports the hypothesis of two different active sources.^52^ Spike localization via the coherent MEM^57^, ^58^ may cancel the aforesaid confounding factor; yet, we chose dipole modeling, being the only clinically approved method, in order to facilitate clinical translation of our findings.

### Ripples‐alone are likely Generated by Non‐Epileptogenic Areas

In case of postoperative good outcome, the brain areas that were not resected were not part of the EZ. Our data from good outcome patients revealed that scalp‐recorded ripples‐alone, which constituted a small portion (16–33%) of all ripples, are very likely (>86% of chance) to be localized in areas that were spared during surgery (not resected or unsampled by icEEG), conversely to ripples‐on‐spikes (34–49% of chance). In these patients, we also observed that the generators of ripples‐alone were the farthest from resection (ESI: 24 mm; MSI: 33 mm), compared to ripples‐on‐spikes (0 mm) or any type of spikes (2–8 mm). Hence, scalp‐recorded ripples‐alone are likely to reflect physiological mechanisms not linked to epileptogenicity. Furthermore, for ripples‐on‐spikes, a larger proportion of their sources was resected in good‐ than poor outcome patients; conversely this was not found for ripples‐alone, suggesting no association between removal of the latter and outcome. In summary, our data indicate that if we only chase ripples‐on‐spikes, physiological counterparts are possibly spared. This has important clinical implications since it alleviates the burden of a noninvasive ripple analyses blinded to spike detection.

### Scalp‐recorded Ripples‐on‐Spikes are Prognostic Biomarkers of Epileptogenicity

Before we translate an epilepsy biomarker to clinical practice for guiding surgery, we must establish whether it can localize the brain tissue that should be resected to yield seizure freedom. Here, we showed that the temporal overlap between ripples and spikes on scalp recordings helps identify the most prognostic epilepsy biomarkers. Removing the cortical areas that generate ripples‐on‐spikes on HD‐EEG or MEG led to good outcome with an accuracy of 86‐90%, prompting the clinical value of noninvasive ripple source imaging in individualized patient care for guiding surgery. Conversely, missed resection of the tissue generating scalp‐recorded ripples‐alone was not associated with outcome. These results reveal that the proposed categorization of scalp‐recorded ripples (based on spike overlap) enhances their prognostic relevance during the presurgical workup. Our study confirms recent MEG findings^25^ and adds to them by demonstrating the predictive value of HD‐EEG ripples and the low localization value of scalp ripples‐alone (on MEG or HD‐EEG).

Furthermore, we investigated the prognostic value of spike localization after distinction between spikes‐on‐ripples and spikes‐alone. Engel and colleagues^59^ suggested that the concurrence with ripples helps discriminate clinically important “red” spikes from nonspecific “green” spikes. Our HD‐EEG findings corroborate this hypothesis: localizing spikes‐on‐ripples predicted outcome (accuracy: 87%), while localizing spikes‐alone did not. For MEG, spikes‐on‐ripples performed better than spikes‐alone (accuracy: 82% vs. 67%), despite the fact that the association between resection and outcome did not reach significance. This result should be attributable to the low sensitivity of MEG to ripples and thus to spikes‐on‐ripple, rather than to a higher localization error, given the similar spatial resolution of HD‐EEG and MEG.^60^, ^61^

Our results can be interpreted in light of in vivo computational models,^62^ which suggested that spikes may also result from transient highly synchronous excitatory inputs in scarcely epileptogenic areas, called “irritative,” which cannot generate seizures. Moreover, spikes‐alone were linked to epileptogenicity more than ripples‐alone (higher prognostic value and shorter resection distance); this suggests that, while the epileptogenicity of spikes suffers from less pathological (irritative) counterparts, ripples are affected by physiological counterparts.

### Limitations

Our cohort presented different etiologies. Given the small sample size, we could not adjust for heterogeneity in outcome prediction; larger prospective studies are needed to this purpose and to validate our predictive model. A minimum 2‐year postoperative follow‐up would have been more ideal. Moreover, our sample did not include patients who: (1) underwent surgery without long‐term intracranial monitoring; (2) did not have presurgical HD‐EEG/MEG recordings; or (3) had icEEG monitoring with depth electrodes solely. This selection bias implicates that our findings may be limited to patients with neocortical epilepsy (not requiring depth electrodes), in whom the EZ estimation was complicated enough to demand for HD‐EEG/MEG recordings and long‐term icEEG. Furthermore, icEEG was not simultaneous to HD‐EEG/MEG, which impeded investigating the exact correspondence between noninvasive source imaging and icEEG. Finally, since children did not always sleep during HD‐EEG/MEG recordings, we did not differentiate sleep from wakefulness. Further studies are encouraged to examine whether spatial specificity of HD‐EEG and MEG ripples to epileptogenic tissue is independent from vigilance state, as shown on icEEG.^63^, ^64^

## Conclusion

We showed that the ripple cortical generators can be localized using scalp HD‐EEG and MEG with high precision compared to intracranial gold standard. Scalp‐recorded ripples‐on‐spikes are prognostic, nonredundant biomarkers of the EZ in children with refractory epilepsy, since their resection leads to good outcome. Conversely, scalp‐recorded ripples‐alone appear to reflect physiological events generated by non‐epileptogenic areas. The possibility to localize prognostic biomarkers of epileptogenicity through noninvasive full‐head techniques would augment presurgical evaluation of children with refractory epilepsy by guiding icEEG implantation and facilitating prognosis. Although our findings indicate that scalp‐recorded ripples may be a presurgical asset, their low detectability represents the prime challenge, which demands for longer recordings, optimized protocols, and instrumentation for high‐frequency recordings.

## Conflict of interests

Nothing to report.
